# Botanical Impurities in the Supply Chain: A New Allergenic Risk Exacerbated by Geopolitical Challenges

**DOI:** 10.3390/nu16050628

**Published:** 2024-02-24

**Authors:** Giulio Dinardo, Lamia Dahdah, Arianna Cafarotti, Stefania Arasi, Vincenzo Fierro, Valentina Pecora, Carmen Mazzuca, Sara Urbani, Maria Cristina Artesani, Carla Riccardi, Rocco Luigi Valluzzi, Cristiana Indolfi, Michele Miraglia del Giudice, Alessandro Fiocchi

**Affiliations:** 1 Department of Woman, Child and of General and Specialized Surgery, University of Campania Luigi Vanvitelli, 80138 Naples, Italy; cristianaind@hotmail.com (C.I.); michele.miragliadelgiudice@unicampania.it (M.M.d.G.); 2 Allergy Diseases Research Area, Pediatric Allergology Unit, Bambino Gesù Children’s Hospital IRCCS, 0165 Rome, Italy; lamiaantanios.dahdah@opbg.net (L.D.); arianna.cafarotti@opbg.net (A.C.); stefania.arasi@opbg.net (S.A.); vincenzo.fierro@opbg.net (V.F.); valentina.pecora@opbg.net (V.P.); carmen.mazzuca@opbg.net (C.M.); sara.urbani@opbg.net (S.U.); mariac.artesani@opbg.net (M.C.A.); carla.riccardi@opbg.net (C.R.); roccoluigi.valluzzi@opbg.net (R.L.V.)

**Keywords:** food allergy, cross-contamination, botanical impurities, vegetable contaminants, food labelling, pediatric, nutrition, reference dose, food safety, public health

## Abstract

Background: The supply chains of food raw materials have recently been heavily influenced by geopolitical events. Products that came from, or transited through, areas currently in conflict are now preferentially supplied from alternative areas. These changes may entail risks for food safety. Methods: We review the potential allergenicity of botanical impurities, specifically vegetable contaminants, with particular attention to the contamination of vegetable oils. We delve into the diverse types of botanical impurities, their sources, and the associated allergenic potential. Our analysis encompasses an evaluation of the regulatory framework governing botanical impurities in food labeling. Results: Unintended plant-derived contaminants may manifest in raw materials during various stages of food production, processing, or storage, posing a risk of allergic reactions for individuals with established food allergies. Issues may arise from natural occurrence, cross-contamination in the supply chain, and contamination at during production. The food and food service industries are responsible for providing and preparing foods that are safe for people with food allergies: we address the challenges inherent in risk assessment of botanical impurities. Conclusions: The presence of botanical impurities emerges as a significant risk factor for food allergies in the 2020s. We advocate for regulatory authorities to fortify labeling requirements and develop robust risk assessment tools. These measures are necessary to enhance consumer awareness regarding the potential risks posed by these contaminants.

## 1. Introduction

With the global changes arising from the COVID-19 pandemic and ongoing world geopolitical tensions, the prices of several vegetable supplies have grown. Recent conflicts are impacting various regions in the European continent, some of which are major suppliers of sunflower oils and cereals worldwide [1]. Russian and Ukrainian territories have emerged as the leading global producers of various agricultural commodities, notably cereals and sunflowers, solidifying the region’s status as the world’s breadbasket [2]. Together, they commanded a staggering 72.7% share of the global sunflower oil and seed trade in 2020 [3,4]. Additionally, approximately 50 countries depend on either Russia or Ukraine for at least 30% of their wheat imports. The ongoing conflict has led to a dramatic reduction in grain and sunflower exports from both Russia and Ukraine. Furthermore, the cost of fertilizers has doubled, while energy prices have increased exponentially [3]. This has led to an abrupt global shortage of sunflower oil, prompting food processors to seek alternative oils. For instance, in India, grains are grown and handled alongside other plants, including peanuts. These alternative products come from different areas of the planet with supply chains that involve different ways of food production, processing, storage, and transport logistics [2]. The presence of these modifications in the supply chain can pose a risk to the allergic population. People with food allergies must follow strict and specific diets to prevent having allergic reactions [5]. However, the existence of “hidden” allergens in foods might undermine patients’ best attempts to establish safe diets [6]. In the present situation, the spotlight has increasingly turned towards botanical impurities, a category of contaminants originating from inadvertent plant-derived sources [6,7]. Unlike intentional ingredients, these impurities can infiltrate various food products at different points along the supply chain, raising concerns about the allergenicity and potential health risks they may pose [8]. Vegetable impurities, as a subset of botanical contaminants, encompass a broad range of plant-derived substances that inadvertently find their way into the final food products [9]. These contaminants can arise from cross-contamination during harvest, transport, and storage, the presence of impurities in raw materials, or natural occurrences in which specific panallergens are inherently incorporated into the food matrix of fruits and vegetables [8]. The importance of addressing the issue of botanical impurities is underscored by its direct correlation with the increasing prevalence of food allergies and associated health risks [10]. Following recent geopolitical upheavals that have shifted trade flows worldwide and consequently altered the supply routes of vegetable raw materials, the issue of contamination within supply chains has become notably pronounced [1,2]. Examples include peanut contamination of soy lecithin and peanut contamination of cocoa [11,12]. Understanding the diverse pathways through which botanical impurities manifest is crucial for food safety campaigns and implementing effective preventive measures and regulatory controls. The incidence of cross-contamination as a source of inadvertent allergic food intake is unclear [8]. People with food allergies can respond to trace levels of the offending food, while there is a wide range of threshold dosages across food allergy groups [13,14,15]. Allergic reactions to these impurities can be varied, ranging from mild symptoms such as itching and hives to severe anaphylactic responses. Epidemiological studies and case series quantify this risk at 20–40% of people suffering from allergies, so reactions to food traces is not uncommon [13,16]. The potential life-threatening nature of these reactions necessitates a comprehensive examination of the allergenic potential of botanical impurities to safeguard public health. Instances of severe allergic reactions can lead to increased healthcare costs, decreased workforce productivity, and emotional distress for affected individuals and their families [17]. Against this backdrop, this study aims to comprehensively explore botanical impurities in food. Specifically, we aim to understand their allergenic properties, origins, and associated risks to consumers. Furthermore, we seek to highlight limitations in current regulatory frameworks and advocate for enhanced labeling requirements. Additionally, we aim to underscore the need for the development and implementation of robust risk assessment tools. In doing so, we aspire to contribute valuable insights to both consumers and regulatory authorities, fostering a safer and more transparent food environment.

## 2. Sensitization Rates of Vegetable Contaminants Potential Allergens

To provide a comprehensive understanding, we begin with a detailed examination of sensitization rates for key vegetable foods. The EuroPrevall evaluation serves as a valuable resource, offering insights into the prevalence of sensitization for each food item (Table 1) [18]. 

### 2.1. Cereals Containing Gluten

These products come from different areas of the planet, such as India, where they are grown and handled in the presence of other plants, including the rachis wheat, which stands out as the most extensively consumed grain globally. Allergic reactions to wheat can manifest as typical food allergies affecting the skin, gastrointestinal tract, or respiratory system. These reactions may also include food-dependent, exercise-induced anaphylaxis (FDEIA); occupational asthma, commonly known as baker’s asthma; rhinitis; as well as contact urticaria. Additionally, the ingestion of wheat gluten has the potential to induce celiac disease, characterized by T cell-mediated inflammation in the intestines, or dermatitis herpetiformis, a skin condition marked by blistering eruptions [19]. Except for the latter two conditions, IgE antibodies play a pivotal role in most of the aforementioned reactions. In IgE-mediated responses, contaminants can trigger clinical reactions. Numerous population-based studies have been conducted to evaluate the prevalence of wheat allergies and sensitization. For instance, a Stockholm-based population cohort (BAMSE) reported a 4% prevalence of sensitization to wheat among 2336 four-year-old children [20]. The German Multi-Centre Allergy Study (MAS), which analyzed longitudinal data from 273 children aged 2 to 10 years, revealed a progressive increase in the prevalence of IgE to wheat, ranging from 2 to 9% [21]. It was suggested that IgE sensitization to wheat primarily occurs in early infancy, often secondary to pollen sensitization at school age. In a random-digit-dial survey by the US Food and Drug Administration (FDA), the self-reported prevalence of doctor-diagnosed wheat allergies among adults in the United States was 0.4% [22]. The pan-European EuroPrevall evaluation indicated sensitization rates ranging from 3.11 (Reykjavik) to 14.44 (Zurich) for wheat and 1.80 (Reykjavik) to 8.35 (Zurich) for corn [18]. Typically, children tend to outgrow wheat allergies, with approximately 65% experiencing resolution by the age of 12 [23]. Certain cereals within the *Poaceae* family, botanically related to wheat, such as rye, oats, barley, corn (maize), and rice, may also elicit allergic reactions [24]. While some are staple foods in many countries, others like millet or kamut are consumed in Europe as a “healthy” alternative or a gluten-free substitute for wheat. Nevertheless, food allergies to these cereals, including severe anaphylaxis, are predominantly recognized in industrialized countries. This is attributed to sensitization occurring primarily through the respiratory route, such as exposure to millet-containing birdseed among pet bird owners [24].

### 2.2. Peanuts

While the peanut allergy may not rank among the most common allergies globally, its significance in shaping the perception of food allergies is substantial. This prominence is primarily attributed to its prevalence in English-speaking nations, particularly the United Kingdom, United States, Australia, Canada, and New Zealand [25]. In these countries, estimates supported by the Oral Food Challenge (OFC) indicate that the frequency of the peanut allergy can reach 2% of the general population—a percentage significantly higher than that observed in most other nations [25]. The impact of the peanut allergy is more pronounced in North America, where it frequently leads to severe anaphylaxis, while its occurrence in Europe is notably lower [26]. In the Asian continent, the peanut allergy is essentially considered a negligible allergen [26]. In Europe, the prevalence of peanut sensitization varies, ranging from 2.31 (Reykjavik) to 10.06 (Zurich) [18]. The reasons behind these regional differences remain not entirely understood. However, understanding the true frequency of peanut allergies is crucial for various markets. It is worth noting that the perception of the severity and prevalence of the peanut allergy may be inflated by certain stakeholders, including parent associations or producers of specific immunotherapy for peanuts. This emphasizes the importance of accurate information and awareness to avoid misconceptions surrounding the gravity and prevalence of the peanut allergy in different regions [26].

### 2.3. Soybeans, Lupin

Numerous studies examining the prevalence of the soy allergy in diverse populations, including infants who are initially fed soy-based formulas, consistently affirm its low allergenicity, despite its notable immunogenicity [27]. The pan-European EuroPrevall assessment has presented sensitization rates ranging from 2.11 (Reykjavik) to 7.99 (Zurich) for lentils and from 1.19 (Reykjavik) to 7.72 (Zurich) for soybeans [18]. Authors have extensively documented cross-reactivity among various legumes and between legumes and different vegetables [28]. This phenomenon arises from the presence of proteins acting as panallergens across various plant species. One such panallergen is Profilin, implicated in cross-reactivity between fruits, vegetables, and certain pollens. Additionally, PR-10 and non-specific lipid transfer proteins (nsLTPs) are becoming increasingly recognized panallergens [29]. Legumes, in particular, demonstrate a considerable degree of cross-sensitization that does not always manifest as clinical reactivity. It is noteworthy that up to 94% of skin prick tests (SPTs) for soybeans have produced false-positive results, and sensitization to carob seeds does not necessarily translate into cross-reactivity among children allergic to peanuts [30].

Despite these nuances, allergic reactions induced by legumes can be potentially severe and, at times, fatal. Such incidents can significantly impact the quality of life for affected patients and their families [31]. Hence, understanding the complexities of cross-reactivity and potential clinical outcomes is crucial in managing legume-related allergies.

### 2.4. Celery

The pan-European EuroPrevall assessment revealed sensitization rates ranging from 2.42 (Reykjavik) to 13.09 (Zurich) for celery [18]. While the celery allergy poses a low risk in countries across the Mediterranean region, instances of anaphylactic reactions have been documented following the ingestion of celeriac, particularly in central Europe [18]. In this context, celeriac transforms into a potentially hazardous allergen, underscoring the geographical variability in the severity of allergic reactions associated with celery consumption.

### 2.5. Sesame Seeds

The sensitization rates for sesame seeds vary from 2.86 (Reykjavik) to 12.10 (Zurich) [18]. Sesame seeds belong to the category of tree nuts, sharing a set of allergens with this group. Noteworthy allergic reactions have been documented, and this allergen is not unheard of in cases of food allergy-related fatalities.

### 2.6. Mushrooms

Mushrooms represent the reproductive structures of macrofungi, primarily falling within the *Basidiomycotina* and *Ascomycotina* divisions. With approximately 14,000 identified species, these fungi release spores from gills or pores on the mushroom caps onto the surface of a substrate [32]. Mycelia, intricate networks of branching hyphae, extract nutrients from the substrate and give rise to fruiting bodies. Edible mushrooms are widely cultivated and consumed across various cultures, both raw and cooked, and are infrequently associated with IgE-mediated hypersensitivity reactions [32].

### 2.7. Yams (Dioscorea *spp.*)

Yams, specifically *Dioscorea opposita*, are commonly consumed in East Asia, yet allergic reactions to this plant food are rare. A few cases have been reported, including instances of anaphylaxis after consuming boiled yams and cases of oral allergy syndrome [33].

### 2.8. Buckwheat (Fagopyrum esculentum *L.*)

Sensitization rates to buckwheat in Europe range from 1.37 (Reykjavik) to 8.89 (Zurich) [18]. Buckwheat is known to cause IgE-mediated allergies, including severe allergic reactions and anaphylaxis [18]. The prevalence of the buckwheat allergy is estimated at 0.1–0.4% in certain Asian regions [34]. Allergy clinic studies indicate a confirmed buckwheat allergy in 2–7% of patients from various countries. Incidences of buckwheat-related anaphylaxis in school studies range from 4 to 60 cases per 100,000 school children. The overall incidence of severe allergic reactions to buckwheat, including anaphylaxis, is estimated at 0.1–0.01 cases per 100,000 person years [34].

### 2.9. Tomatoes

In Europe, sensitization rates to tomatoes range from 2.55 (Reykjavik) to 13.27 (Zurich) [18]. The tomato allergy is less frequent, but immediate-type reactions are well-documented, especially in atopic individuals. Clinical symptoms occur in 2.3% to 40% of patients with food allergies, with a prevalence of 6.5% in the Mediterranean population attending allergy clinics [35].

### 2.10. Sunflower Seeds and Products Thereof

The European sensitization rate for sunflowers is 1.37 (Reykjavik) to 8.89 (Zurich) [18].

In conclusion, all the botanical species mentioned have the potential to trigger allergies. However, the severity of the risk varies among species and is not uniform across different geographical locations.

## 3. Characterization of Vegetable Allergens: Unveiling Molecular Complexity 

The allergens associated with the vegetable foods analyzed in this review are cataloged in the Pfam database, a comprehensive collection of protein families represented by multiple sequence alignments and hidden Markov models (HMMs) [36]. The current version encompasses 16,295 protein families, organized into superfamilies referred to as clans in Pfam (e.g., the prolamin superfamily). Certain families are further subdivided into subfamilies, such as the PR-10-like proteins subfamily within the Bet v 1 family [36].

A protein family constitutes a set of proteins sharing a common evolutionary origin, primarily reflected in their similar overall structure and topology. Members of a protein family may exhibit related biological functions, immunologic characteristics, and similar amino acid sequences. This suggests that certain protein families are shared among various botanical families, and consuming them could result in instances of cross-reactivity, leading to allergic reactions with different types of vegetable foods [36].

Importantly, regarding the topic of contamination, some of these allergens are thermostable and resistant to digestion. There are four superfamilies of interest regarding our topic of focus (Table 2).

### 3.1. Prolamin Superfamily

The prolamin superfamily comprises proteins identified by the existence of a globular domain with an alpha-helical structure. This domain features a consistent arrangement of cysteine residues, which create three to five intra-molecular disulfide bonds. The prolamin superfamily encompasses various groups, such as cereal prolamin seed storage proteins (gliadins and glutenins), as well as several families of small proteins rich in disulfide bonds, including bifunctional inhibitors, 2S albumin seed storage proteins, and non-specific lipid transfer proteins [29].

#### 3.1.1. Cereal Prolamins

Exclusive to the grains of cereal grasses, seed storage proteins known as cereal prolamins exhibit a distinct characteristic. Unlike their low-molecular-weight counterparts in the superfamily, the alpha-helical domain of cereal prolamins experiences disruption due to the insertion of repetitive sequences. Gliadins and glutenins are representative members of the cereal prolamin family. Wheat, in particular, contains various allergenic cereal prolamins, such as Tri a 19, an x-5 gliadin, Tri a 21, an a/b gliadin, and Tri a 26, a high-molecular-weight glutenin [29].

#### 3.1.2. Bifunctional Inhibitors

Similar to cereal prolamins, bifunctional inhibitors are exclusively found in cereal grains. These allergens can induce sensitization either through the respiratory tract, via the inhalation of flour, or through the gastrointestinal tract by consuming foods containing wheat, barley, or rice. Bifunctional inhibitors are proteins ranging from 12 to 16 kDa, held together by 4–6 disulfide bonds [29,37]. Their structural forms include monomeric, heterodimeric, and heterotetrameric, depending on the degree of aggregation among their subunits. They are a significant cause of baker’s asthma and also contribute to plant/food allergies [38]. Notable examples include the following:

- Hor v 15, a monomeric α-amylase inhibitor from barley.
- Tri a 28, a dimeric α-amylase inhibitor, and Tri a 29, a tetrameric α-amylase inhibitor, both from wheat.

#### 3.1.3. 2S Albumins

The 2S albumins represent a significant category among plant seed storage proteins. Typically, most 2S albumins are initially produced as single-chain proteins that undergo subsequent cleavage into a small and large subunit. Both subunits form compact alpha-helical structures through 4–5 disulfide bonds. Notably, several crucial seed and tree nut allergens belong to the 2S albumin group [29].

Some allergenic 2S albumins include the following:

- Ara h 2 and Ara h 6 from peanuts.
- Ber e 1 from Brazil nuts.
- Cor a 14 from hazelnuts.
- Jug r 1 from English walnuts.
- Ses I 1 from sesame seeds.
- Sin a 1 from yellow mustard.

The families of seed storage proteins serve as primary triggers for IgE sensitization to tree nuts, legumes, and seeds. These allergens possess heat stability and might be accountable for severe, potentially fatal adverse reactions to these particular foods [29].

#### 3.1.4. Non-Specific Lipid Transfer Proteins 

nsLTPs have been proposed to facilitate the transfer of phospholipids between vesicles and membranes. However, plants utilize the three-dimensional structures of nsLTPs in diverse ways, with many nsLTPs playing a crucial role in plant defense against fungi and bacteria [39]. Allergenic nsLTPs form a substantial group of proteins known for their resistance to heat and digestion. They are abundantly present in the epidermal tissues of fruits and serve as major allergens in fruits from the *Rosaceae* family. Additionally, allergenic nsLTPs are found in nuts, seeds, vegetables, and *Hevea brasiliensis* latex [29,40].

Examples of nsLTPs in *Rosaceae* fruits include the following:

- Mal d 3 from apples.
- Pru p 3 from peaches.

Representative allergenic nsLTPs from tree nuts include the following:

- Cor a 8 from hazelnuts.
- Jug r 3 from walnuts.

Sensitization to nsLTPs is linked to a higher likelihood of subjects experiencing systemic symptoms, and it is not limited to oral allergy symptoms. Furthermore, allergic reactions may occur upon ingesting various fruits, vegetables, and legumes, including those from the *Rosaceae* family, as well as celery, carrots, walnuts, fennel, peanuts, cereals, and tomatoes [40,41].

### 3.2. Profilin-Like Superfamily

Profilins are intracellular proteins present in eukaryotic cells. They interact with monomeric actin and various muscle proteins, playing a crucial role in regulating the dynamics of actin polymerization during cellular processes such as cell movement, cytokinesis, and signaling. In higher plants, profilins form a highly conserved protein family with sequence identities of at least 75%, even among members from distantly related organisms [29,42]. Profilins from higher plants are known to bind to monomeric actin and contribute to the regulation of actin dynamics. Interestingly, sequence conservation extends across various plant species. Sensitization to profilins has been considered a risk factor for pollen-associated food allergies, as IgE specific to profilins often cross-reacts with homologs from most plant sources [42].

Some allergenic profilins include the following:

- Cit s 2 from oranges.
- Cuc m 2 from melons.
- Mus a 1 from bananas.

### 3.3. Cupin Superfamily 

Cupins represent an extensive superfamily of proteins with a shared origin, and their evolutionary history can be traced from bacteria to eukaryotes, encompassing animals and higher plants. Currently, cupin proteins are categorized into 57 protein families within this superfamily. Notably, the largest families of bicupins include the 7/8S and 11S seed storage globulins, which serve as the predominant components of plant seeds. These globulins are not only vital sources of proteins for the human diet but also notable allergens [29,43]. Of particular interest are the families of vicilins and legumins. 

#### 3.3.1. Vicilins

Homotrimeric proteins typically have a molecular weight ranging from 150 to 190 kDa. Their specific subunit compositions can vary significantly and is influenced by the differences in proteolytic processing and glycosylation of the individual monomers [29]. Some allergenic vicilins, belonging to the homotrimeric protein category, include the following:

- Ara h 1 from peanuts.
- Gly m 5 from soybeans.
- Jug r 2 from walnuts.
- Ses I 3 from sesame seeds.

#### 3.3.2. Legumins

In mature legumins, two trimers combine to create hexameric proteins [29]. Noteworthy allergenic legumins within this category include the following:

- Ara h 3 from peanuts.
- Gly m 6 from soybeans.
- Ber e 2 from Brazil nuts.
- Fag e 1 from buckwheat.

### 3.4. Bet v 1-Like Superfamily

**Figure 1:**
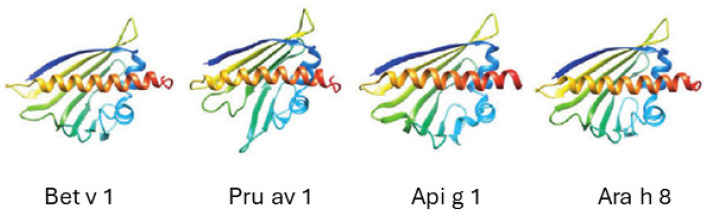
Four representative members of the Bet v 1 family of proteins. Bet v 1 is from birch pollen, Pru av 1 from cherries, Api g 1 from celery, and Ara h 8 from peanuts.

The Bet v 1-like superfamily encompasses 14,065 proteins derived from 1452 species, all of which share sequences related to the major birch (*Betula verrucosa*) pollen allergen Bet v 1. These proteins are distributed across 14 families, with each family, including the Bet v 1 family, characterized by the same structural features: 7 antiparallel beta-sheets and 3 alpha-helices. Their structure also includes a cavity between the beta-sheets and a lengthy C-terminal alpha-helix. This cavity has the potential to bind various lipid and bioflavonoid molecules. Of the 14 families within the Bet v 1-like superfamily, allergens have been identified in 3 of the 11 subfamilies of the Bet v 1 family. However, only one of these subfamilies is of interest for the present topic (Figure 1) [29].

#### The PR-10 Proteins

Within the Bet v 1 family, 1 of the 11 subfamilies consists of proteins known as PR-10 proteins. The expression of these proteins is influenced by factors such as pathogenic attacks, abiotic stress, or developmental regulation. PR-10 proteins are notably abundant in reproductive tissues like pollen, seeds, and fruits [29].

Many individuals allergic to birch pollen may experience allergic reactions to various fruits and vegetables due to IgE cross-reactivity between Bet v 1 and the homologous allergens found in plant foods. Most Bet v 1-related food allergens are identified in certain plant families, including *Rosaceae* (apple, pear, stone fruits), *Apiaceae* (celery, carrot), and *Fabaceae* (soybean, peanut). Sensitization to PR-10-like proteins and profilins, without nsLTP, is associated with oral allergy syndrome rather than systemic reactions [29].

Given this context, each of the foods mentioned as potential vegetable contaminants carries several vegetable allergens, particularly the following.

### 3.5. Cereals Containing Gluten

Several clearly identified allergens contribute to the broad cross-reactivity observed among them. These allergens include alpha-amylase/trypsin inhibitors, LMW glutenins, and nsLTPs found in rice, corn, wheat, and barley. There is also a potential association with millet allergy in certain cases. Wheat, in particular, contains a minimum of 15 distinct food allergens [29].

### 3.6. Peanuts

Several peanut allergens have been characterized, with many of them serving protective functions or acting as seed storage proteins. These peanut allergens are part of various protein families, fostering IgE-mediated cross-reactions in immune responses. These cross-reactions extend not only among different members of the legume families but also involve other plant foods, including tree nuts [44]. The toasting process seems to increase the allergenicity of key peanut allergens [45,46]. Peanut allergens’ stability enables them to be captured by ventilation filters. Incidents of allergic reactions resulting from the inhalation of peanut particles on airplanes and in schools have been documented [47,48,49].

### 3.7. Soybeans, Lupin

The allergenic properties of soybeans can be influenced by technological treatments, and the impact of cooking on these proteins is not consistently interpreted [50]. Storage and heat processing play a role in altering the in vitro allergenicity of soybeans, with a microwave treatment of 25 min showing a slight reduction in allergenicity [51,52]. Legumes, in general, exhibit a significant degree of cross-sensitization, but this does not always translate into clinical reactivity. False-positive results for soybeans have been reported in up to 94% of SPT, and sensitization to carob seeds does not necessarily result in cross-reactivity among children allergic to peanuts [30,53]. These observations have clinical implications. For instance, carob may be considered acceptable as an additive in processed foods and formulas, as avoidance of its proteins is not recommended based on the low cross-reactivity observed within the *Leguminosae* family. At least 16 soybean allergens have been identified, displaying metabolic, storage, or protective functions across diverse protein families with conserved three-dimensional structures [54]. This leads to extensive IgE-mediated cross-reactions among different members of the legume families and other plant foods. Due to the complexity and heterogeneity of soybean proteins, the relationship between their structure and allergenicity has only been partially addressed. Over the recent two decades, additional soy allergens (Gly m 4–8) have been identified and officially accepted by the IUIS Allergen Nomenclature Sub-Committee. However, reliable data on sensitization rates are currently available only for Gly m 4, 5, and 6 [55].

### 3.8. Sesame Seeds 

Sesame is characterized by at least five allergens, some of which exhibit thermostability. The main allergens include Ses I 1 and Ses I 2 [29].

### 3.9. Mushrooms

Mushrooms are rarely implied in food allergy and their allergens are not well-characterized [32]. 

### 3.10. Yams (Dioscorea *spp.*)

In immunoblotting experiments with sera from patients allergic to yams, a 30-kDa protein was consistently recognized by all patients, while a 17-kDa band was specifically detected by one patient. The N-terminal amino acid sequencing revealed that the 30-kDa IgE-reactive band was identified as DB3S, which stands for dioscorin in the *Dioscorea* tuber. This discovery suggested that DB3S is a thermally stable oral allergen capable of triggering anaphylactic reactions and oral allergy syndrome in individuals with cooked yam (*Dioscorea opposita*) allergies [29,33].

### 3.11. Buckwheat (Fagopyrum esculentum *L.*)

Several allergenic proteins have been identified in common buckwheat, including Fag e 1, Fag e 2, and Fag e 3. In Tartary buckwheat, proteins such as Fag t 1, Fag t 2, and Fag t 3 have been identified. Clinically relevant cross-reactivity has been observed between buckwheat and other allergenic sources, such as peanuts, latex, coconuts, quinoa, and poppy seeds [29,34].

### 3.12. Celery and Tomatoes

Tomatoes (*Solanum lycopersicum*) share several allergens with celery and carrot, including profilins, PR-10, LTP. Tomatoes own specific allergens, whose thermostability is not high [29,35].

### 3.13. Sunflower Seeds and Products Thereof

The most known allergen of *Helianthus annuus* (sunflower) is Hel a 1, a defensin-like protein. Reactions to it may be anaphylactic [29].

## 4. The Regulatory Context

**Figure 2:**
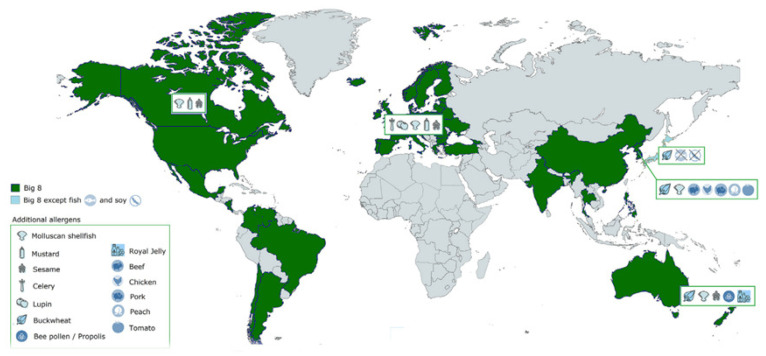
Examples of allergies subject to obligatory allergy labeling across the world. The eight main allergies, or “The Big 8,” are indicated in shades of green for the nations where they are present and need to be notified. Country-specific allergies that are also required to be labeled are displayed with specialized icons. (Adapted from Fiocchi et al., 2021) [67].

The responsibility for ensuring the safety and accurate labeling of food lies with those involved in its production and sale [56]. The food industry, in its pursuit of delivering safe products and catering to consumer preferences, must institute robust policies and procedures to ensure precise product labeling and mitigate the potential for allergen cross-contamination at every stage of the food supply chain—ranging from harvesting, storage, and transportation to processing and equipment cleaning [56]. In the EU Regulation 2021/382, there is a focus on managing food allergens [57]. This regulation not only introduces the concept of ‘food safety culture’ but also establishes new requirements for food redistribution in accordance with Good Manufacturing Practice (GMP) principles. These updates stem from reforms initiated in September 2020 by the Codex Alimentarius Commission, resulting in revisions to the Hygiene Package [58]. These updates emphasizes allergen management, urging all stakeholders in the food supply chain to implement self-control measures to mitigate the risk of allergen contamination. Secondly, it aims to foster a culture of food safety awareness and training among all workers involved in food handling beyond the primary agricultural sector. Lastly, specific hygiene and traceability requirements are established for food redistribution activities, ensuring food safety even in charitable endeavors [57,59]. Labelling is an issue of relevance to consumers with food allergies with regard to processed or pre-packaged foods, as accidental ingestion of allergens in pre-packaged processed foods, due to labelling ambiguities, is a modifiable risk factor [60]. In the European Union, until 2005, legislators allowed composite ingredients to be listed on labels if not in excess of 25 per cent of the whole product. Thus, a snack may have mentioned having “mayonnaise” on the label without explicitly indicating eggs as an ingredient [61]. This clause was eliminated in 2005, following the review of a labelling directive issued in September 2001 [62]. Twelve food items are now required by law to appear on the label: cereals containing gluten, crustaceans, eggs, fish, peanuts, soy, milk, nuts, mustard, sesame seeds, celery, and sulfites > 10 mg/kg [60,63,64]. A similar legislation is in effect in the US. Starting from January 2006, the Food Allergen Labeling and Consumer Protection Act provides that all food products require an ingredient statement [65]. This has altered industry practices in some important respects for milk, eggs, peanuts, tree nuts, shellfish, fish, soy, wheat, and sesame seeds (Figure 2) [66].

**Figure 3:**
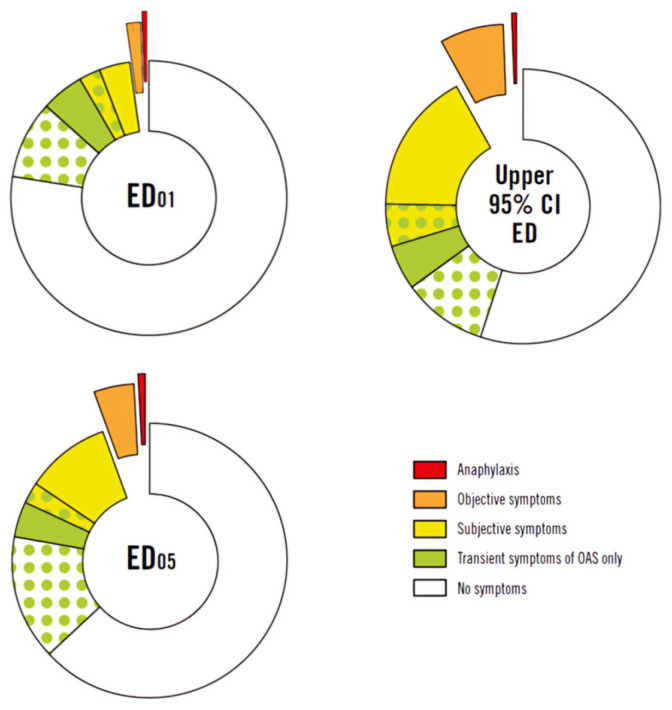
Objective symptoms after exposure to an ED01 or ED05 amount of peanuts. OAS = oral allergy symptoms. ED01, the eliciting dose predicted to provoke reactions in 1% of the allergic population; ED05, the eliciting dose predicted to provoke reactions in 5% of the allergic population. (Adapted from Turner et al., 2022) [69,71].

Thus, hidden allergens previously not requiring labeling because they found in ingredients/additives exempt from specific labeling (i.e., colors and flavorings, etc.) have to be disclosed if they contain one of the nine above-listed foods. Thus, many of the problems with unlabeled hidden allergens in the food supply may be applicable anymore. In particular, the risk that unfamiliar names can hide allergenic foods is now addressed, For example, terms like “starch” will be substituted with more specific labels such as “corn starch” or “wheat starch,” and “Lysozyme” will be replaced with descriptors like “lysozyme, containing egg”, and so on [67]. On both sides of the Atlantic, the regulatory problem is now the opposite concern—whether too many foods containing trace amounts of these allergenic foods are being precautionary “overlabeled” and whether this would then potentially restrict potentially safe food choices for allergic consumers [60]. Actually, the use of precautionary allergen labeling (PAL) falls into a legislative grey area, often being unregulated and non-standardized, with no clear guidelines on when to use or not use it. PAL is frequently utilized on a voluntary basis and without any precise rules, and many manufacturers are now indicating statements such as “may contain...”, “may contain traces of...”, and “produced in a facility that uses...” as a warning of potential contaminations during food preparation [67,68]. The Article 36(3)(a) of Regulation (EU) 1169/2011 provides a legal foundation for requirements on voluntary information on the probable and unavoidable presence in food of impurities causing allergies or intolerances owing to cross-contamination [64]. The regulation makes no mention of a timeframe for enacting this act. As previously stated, there are currently no formal legal definitions of PAL in the EU. Some nations, such as Argentina, prohibit PAL, while others, such as Japan, have made efforts to require labeling unintended allergen presence (UAP) over a detectable level, i.e., 10 ppm [68]. Still, other nations (for example, Belgium and The Netherlands) have proposed reference doses for allergy management based on similar concepts as VITAL^®^ (Voluntary Incidental Trace allergy Labelling) [68]. In recent years, several meta-analyses have been conducted to assess the threshold dose of various foods that allows for a “tolerable risk”. Turner et al. revealed that approximately one-third of individuals with peanut allergies exposed to an ED05 dose (the dose predicted to provoke reactions in 5% of the at-risk allergic population) would experience subjective symptoms, typically mild and short-lived ones. Among the predicted 5% of individuals who would develop objective symptoms, only 4.5% were expected to experience anaphylaxis. The authors concluded that peanuts could be used as an exemplary allergen in terms of characterizing the hazard at ED05 or lower exposure levels [69]. In 2023, the FAO/WHO Codex Committee on Food Labelling proposed thresholds for PAL based on the ED05 values published as the most appropriate reference doses to guarantee the transparency of information to the consumer and safeguard his/her safety (Figure 3) [70,71]. 

## 5. Discussion

In the midst of complex global dynamics, ensuring food safety emerges as a crucial concern intertwined with geopolitical issues, climate change, and economic turbulence. Within this intricate context, attention turns to the lesser-known but equally vital aspects of our food supply: botanical impurities. As the world grapples with geopolitical conflicts, the impact on various facets of the food chain becomes increasingly significant [2]. In this comprehensive analysis of vegetable contaminants and their potential as allergens, we delve into the intricate molecular complexity underlying these allergens and explore the existing regulatory landscape. The investigation reveals that several vegetable contaminants carry allergenic proteins, posing a potential risk for individuals susceptible to allergies. Notably, diverse families of proteins, such as 2S albumins, nsLTPs, profilins, cupins, and Bet v 1-like proteins, were identified across various vegetables. These proteins exhibit heat stability and resistance to digestion, emphasizing their potential to trigger adverse reactions in individuals with susceptible allergies [8,9]. Notably, diverse families of proteins, such as 2S albumins, nsLTPs, profilins, cupins, and Bet v 1-like proteins, were identified across various vegetables. These proteins exhibit heat stability and resistance to digestion, emphasizing their potential to trigger adverse reactions in sensitive individuals. The molecular characterization of vegetable allergens underscores their complexity and diversity. Families such as 2S albumins, nsLTPs, profilins, cupins, and Bet v 1-like proteins exhibit unique structural features, and their presence extends beyond specific vegetables to encompass a broad range of plant sources [29]. Sensitization to these allergens increases the likelihood of systemic symptoms, emphasizing the importance of understanding their structural variations for effective food safety management. The ongoing geopolitical events have influenced the utilization of alternative supply chains for raw materials, such as cereals and sunflower oils. The different supply chain could pose a food safety risk for patients with allergies due to the presence of botanical impurities. On the other hand, the regulatory context surrounding botanical impurities and cross contamination is critical for ensuring food safety and accurate labeling [6]. The responsibility for meticulous product labeling falls on stakeholders across the food supply chain, from harvesting to processing [57]. Recent regulatory changes, both in the European Union and the United States, mandate explicit labeling of major allergens, eliminating ambiguities that might lead to accidental allergen ingestion. However, the use of precautionary allergen labeling (PAL) introduces a new layer of complexity. While regulations like Article 36(3)(a) of Regulation (EU) 1169/2011 provide a legal foundation for voluntary information on allergen impurities, the lack of standardized guidelines raises concerns about potential overlabeling [60]. Different nations adopt varied approaches, with some prohibiting PAL, others requiring labeling of unintended allergen presence, and some proposing reference doses for allergy management. The voluntary nature and the differing approach to the use of PAL by various nations creates confusion and a grey area in which some food industries may choose not to use PAL, while others, conversely, might use it excessively and improperly [72].

## 6. Conclusions

Recent geopolitical tensions have modified the supply routes of vegetable raw materials, highlighting the issues of botanical impurities and food safety. The botanical impurities and cross-contamination of foods with residues of other allergenic foods is possible due to the frequent usage of common facilities and equipment. Foods contaminated with allergenic food contaminations from such procedures may provide a risk to consumers with food allergies. However, whereas persons with food allergies are subjected to unintentional exposures frequently, the fraction of these exposures caused by cross-contamination is unclear. This in-depth exploration of vegetable contaminants as potential allergens highlights the need for continued research, standardized regulations, and increased awareness. While substantial progress has been made in allergen labeling, ongoing efforts are crucial to address the emerging challenges and ensure the safety of individuals with allergies. The findings presented here contribute valuable insights to the broader discussion on vegetable allergens in the context of evolving regulatory frameworks and our scientific understanding. Certain vegetable foods demand allergen declaration, encompassing cereals containing gluten such as wheat, rye, barley, oats, spelt, kamut, or their hybridized strains, along with peanuts, soybeans, celery, sesame seeds, and lupin. Moreover, there are additional food allergens not covered by the EU directive, including mushrooms, yams (*Dioscorea* spp.), buckwheat (*Fagopyrum esculentum* L.), tomatoes, and sunflower seeds and their derivatives. It is imperative to challenge the notion of avoiding foods based on the fear of cross-contamination. Contrarily, the predominant focus in allergy medicine is on preventing the unnecessary elimination of foods. For this reason, a better knowledge of threshold dosages on an individual and population level is required so that quantitative risk assessment may be utilized to guide food industry allergy management efforts, establish their efficacy, and provide a solid foundation for labeling choices.

## Figures and Tables

**Table 1:** Prevalence of food sensitization from Zurich to Reykjavik: insights from the EuroPrevall evaluation [18].

Prevalence (95% CI) of Food Sensitization to:	Reykjavik	Zurich
Wheat	3.11 (2.39–3.82)	14.44 (12.98–15.90)
Hazelnut	1.87 (1.31–2.43)	14.35 (12.89–15.81)
Tomato	2.55 (1.90–3.23)	13.27 (11.86–14.68)
Peach	2.49 (1.84–3.13)	13.21 (11.80–14.62)
Celery	2.42 (1.79–3.06)	13.09 (11.69–14.49)
Carrot	2.11 (1.52–2.71)	12.46 (11.09–13.83)
Sesame seed	2.86 (2.17–3.55)	12.10 (10.74–13.45)
Apple	2.05 (1.46–2.64)	11.95 (10.60–13.30)
Peanut	2.31 (1.69–2.93)	10.06 (8.81–11.31)
Walnut	1.37 (0.89–1.85)	9.52 (8.30–10.74)
Buckwheat	1.37 (0.89–1.85)	8.89 (7.70–10.07)
Sunflower seed	1.37 (0.89–1.85)	8.89 (7.70–10.07)
Poppy seed	0.75 (0.39–1.10)	8.50 (7.34–9.66)
Corn	1.80 (1.25–2.35)	8.35 (7.20–9.50)
Lentils	2.11 (1.52–2.71)	7.99 (6.86–9.11)
Soybean	1.19 (0.74–1.63)	7.72 (6.61–8.83)
Mustard seed	0.37 (0.12–0.63)	4.92 (4.02–5.82)

**Table 2:** Overview of the most important allergen superfamilies and families with their corresponding sources [29].

Superfamily	Family	Allergen Sources
Prolamin	Cereal prolamins	Grains of cereal grasses
	Bifunctional inhibitors	Grains of cereal grasses
	2S albumins	Tree nuts, peanuts, legumes, seeds
	Non-specific lipid Transfer proteins	Fruits, tree nuts, peanuts, vegetables
Profilin-like	Profilin	Fruits, vegetables
Cupin	Vicilins	Tree nuts, peanuts, legumes, seeds
	Legumins	Tree nuts, peanuts, legumes, seeds
Bet v 1-like	PR-10	Fruits, vegetables, legumes, tree nuts
